# Associations between Lifetime Spanking/Slapping and Adolescent Physical and Mental Health and Behavioral Outcomes

**DOI:** 10.1177/07067437211000632

**Published:** 2021-03-09

**Authors:** Janique Fortier, Ashley Stewart-Tufescu, Samantha Salmon, Harriet L. MacMillan, Andrea Gonzalez, Melissa Kimber, Laura Duncan, Tamara Taillieu, Isabel Garces Davila, Shannon Struck, Tracie O. Afifi

**Affiliations:** 1 Department of Community Health Sciences, University of Manitoba, Winnipeg, Manitoba, Canada; 2 Faculty of Social Work, University of Manitoba, Winnipeg, Manitoba, Canada; 3 Department of Psychiatry and Behavioural Neurosciences, McMaster University, Hamilton, Ontario, Canada; 4 Department of Pediatrics, McMaster University, Hamilton, Ontario, Canada; 5 Department of Health Research Methods, Evidence and Impact, McMaster University, Hamilton, Ontario, Canada; 6 Offord Centre for Child Studies, Department of Psychiatry and Behavioural Neurosciences, McMaster University, Hamilton, Ontario, Canada; 7 Department of Psychiatry, University of Manitoba, Winnipeg, Manitoba, Canada

**Keywords:** spanking, physical punishment, mental health, physical health, defiant behaviors, childhood adversities, child maltreatment

## Abstract

**Background:**

Many parents use physical forms of punishment, including spanking to correct perceived misbehavior. While some authors suggest spanking/slapping is a distinct and “milder” form of physical punishment, parents’ use of spanking is consistently associated with poor outcomes for their children. However, less is known about the relationship between spanking/slapping and health and behavioral outcomes in adolescence independent of other childhood adversities.

**Objectives:**

The objectives of this study were to examine the associations between lifetime experiences of spanking on the bottom and/or slapping on the hand and 3 adolescent outcomes: (a) mental health disorders, (b) physical health conditions, and (c) defiant behaviors, after adjusting for other types of childhood adversities and child maltreatment.

**Methods:**

Cross-sectional data from the provincially representative 2014 Ontario Child Health Study (*N* = 6,537 dwellings, response rate = 50.8%) were used. The current study focused on one selected child aged 14 to 17 years within a household (*n* = 1,883) with data collected from the adolescent and the parent/caregiver. Logistic regression models were used to identify associations with lifetime experiences of spanking/slapping 3 or more times (vs. 0 to 2 times).

**Results:**

Lifetime spanking/slapping was independently associated with increased odds of mental health disorders, physical health conditions, and defiant behaviors in adolescence after adjusting for childhood adversities and child maltreatment (unadjusted and adjusted odds ratios ranging from 1.29 to 2.19).

**Conclusions:**

These findings suggest that lifetime spanking/slapping is uniquely associated with harmful mental, physical, and behavioral outcomes in adolescence, and efforts should focus on its prevention.

## Abstract (fr)

**Contexte:**

Plusieurs parents utilisent des formes physiques de punition, notamment la fessée pour corriger une mauvaise conduite perçue. Bien que certains auteurs suggèrent que la fessée/gifle est une forme distincte « plus douce » de châtiment physique, l’usage de la fessée par les parents est constamment associé à de mauvais résultats pour leurs enfants. Cependant, on en sait moins sur la relation entre la fessée/gifle avec la santé et les résultats comportementaux à l’adolescence, indépendamment d’autres adversités au cours de l’enfance.

**Objectifs:**

Les objectifs de la présente étude étaient d’examiner les associations entre les expériences de durée de vie de fessées sur le derrière et/ou de tapes sur la main et trois résultats à l’adolescence: (a) troubles de santé mentale, (b) affections de santé physique, et (c) comportements d’opposition, après ajustement pour d’autres types d’adversités dans l’enfance et la maltraitance des enfants.

**Méthodes:**

Les données transversales de l’Enquête sur la santé des jeunes Ontariens 2014 à l’échelle provinciale (*N* = 6 537 ménages, taux de réponse = 50,8%) ont été utilisées. L’étude actuelle portait sur un enfant sélectionné de 14 à 17 ans au sein d’un ménage (n = 1 883) et des données recueillies auprès de l’adolescent et du parent/soignant. Des modèles de régression logistique ont servi à identifier les associations aux expériences de durée de vie de fessée/gifle de trois fois ou plus (c. 0 à 2 fois).

**Résultats:**

La fessée/gifle de durée de vie était indépendamment associée à des probabilités accrues de troubles de santé mentale, d’affections de santé physique et de comportements d’opposition à l’adolescence, après ajustement pour les adversités de l’enfance et la maltraitance d’un enfant (rapports de cotes non ajustés et ajustés allant de 1,29 à 2,19).

**Conclusions:**

Ces résultats suggèrent que la fessée/gifle de durée de vie est uniquement associée à des résultats mentaux, physiques et comportementaux préjudiciables à l’adolescence et les efforts devraient porter sur la prévention.

Physical or corporal punishment is defined as “any punishment in which physical force is used and intended to cause some degree of pain or discomfort, however light” (para. 11).^1^ One of the most common forms of physical punishment is spanking, often defined as “hitting a child on their buttocks or extremities using an open hand” (p. 453).^2^ In the United States and Canada, estimates of caregivers’ self-reported use of physical punishment ranged from 23% to 80%.^3–7^ An increasing number of international and professional organizations, such as the World Health Organization and the American Academy of Pediatrics, have issued statements in support of ending *all* forms of physical or corporal punishment against children and youth.^8,9^ As of 2020, 60 countries/states have prohibited the use of physical or corporal punishment.^10^ However, this only represents 13% of the global child population,^10^ and physical or corporal punishment is still endorsed and used by many parents.

There remains a perceived dichotomy between physical punishment and abuse, in particular when it comes to spanking that is often perceived as “milder”^11^ or “subabusive.”^12^ One of the common criticisms of the spanking literature is that the severity of some of the forms of physical punishment closely resembles physical abuse.^2^ However, as reflected in the United Nations Committee on the Rights of the Child’s definition of physical or corporal punishment, there are no lines or “thresholds” separating the two^1,12^; in fact, “most child physical abuse occurs in the context of punishment” (p. 1373).^13^ This false dichotomy is further reflected in the literature with researchers using language such as “appropriate” versus “inappropriate,” “abusive” versus “nonabusive,” “reasonable” versus “unreasonable” physical punishment.^11,12^ These perceptions persist despite empirical evidence confirming that spanking is an adverse childhood experience (ACE).^14,15^

This is further reflected in parental beliefs and social norms, particularly as they relate to spanking. In one study, spanking/slapping was the form of physical punishment most frequently used by parents who did not endorse physical punishment.^11^ Furthermore, a recent study demonstrated that adults’ perceptions of physical punishment differed according to the verb used to describe the act: with spanking perceived as “the most common, acceptable, and effective” (p. 5) relative to verbs including “swat,” “hit,” “slap,” and “beat.”^16^ Parents who use spanking express the belief that it will result in positive short- and long-term outcomes for the child.^17,18^

In contrast to these beliefs, there is an extensive and growing literature demonstrating that physical punishment, including spanking, is associated with poor outcomes across the life span, including poor mental health, poor physical health, and problem behaviors.^2,14,19–22^ Spanking may change the structure and function of biological processes and/or promote maladaptive coping behaviors resulting in poor mental and physical health outcomes and problem behaviors.^23^ Research on the association between spanking and problem behaviors are of particular importance, given that spanking is often viewed as a response to and/or a strategy to discourage perceived misbehavior and increase compliance. There are, however, notable limitations and gaps in the literature examining the associations between spanking and poor outcomes.

An important limitation in the literature is the variability in definitions and lack of specificity in the types of physical punishment studied. This lack of specificity could result in underreporting of perceived “milder” forms of physical punishment such as spanking.^11,16^ Past research has been criticized for measuring forms of physical punishment perceived as “more severe” and closely resembling physical abuse, which has led some researchers to suggest that only “more; severe” forms of physical punishment are associated with detrimental outcomes.^11^

Another important evidence gap is the lack of statistical adjustment for other childhood adversities. Physical punishment, including spanking, does not occur in isolation and has been associated with other forms of child maltreatment, including physical abuse, sexual abuse, emotional abuse, emotional neglect, physical neglect, and exposure to intimate partner violence (IPV).^14,24,25^ Therefore, it can be difficult to determine its independent association with harmful outcomes. Some researchers propose that spanking should be considered an ACE, which traditionally includes childhood experiences of abuse and neglect (i.e., physical, sexual, emotional, violence against the child’s mother, physical neglect, and emotional neglect) and household challenges (i.e., a household member with substance use problems, a household member with mental illness, parental separation or divorce, and incarceration of a household member).^14,26^ Similar to physical punishment, these childhood adversities have consistently been associated with poor long-term mental and physical health outcomes.^26–33^ Limited studies on physical punishment have adjusted for only some child maltreatment or adversity variables;^14,25,34,35^ however, many of these examined harsh physical punishment, including practices such as pushing, grabbing, shoving, hitting, or slapping,^20,21,34,36^ which may be criticized because of their level of severity.^2^ One study involving U.S. adults, which looked specifically at the associations between spanking and health and mental health outcomes, only adjusted for physical and emotional abuse.^14^ There is a need to better understand how spanking, a common and often perceived as “milder” form of physical punishment,^11^ is associated with health and behavioral outcomes independent of other, often co-occurring, childhood adversities.

The objectives of the current study were to examine the associations between lifetime spanking/slapping and 3 outcomes in a provincially representative sample of adolescents living in Ontario, Canada: (a) mental health disorders, (b) physical health conditions, and (c) defiant behaviors, while adjusting for sociodemographic variables, childhood adversities, and child maltreatment.

## Methods

### Data and Sample

Data were collected from 2014 to 2015 as part of the 2014 Ontario Child Health Study (2014 OCHS) in Ontario, Canada. The 2014 OCHS involved a provincially representative sample using a 3-stage sampling design to randomly select private households with children aged 4 to 17 years (*N* = 6,537 dwellings, response rate = 50.8%). In the current study, data were collected within each household from 1 randomly selected child and the person most knowledgeable (PMK) about that child, hereinafter referred to as parent/caregiver. The majority of parents/caregivers were biological, adoptive, or stepparents (88.4% mothers, 9.7% fathers), and the remaining 1.4% were related or unrelated caregivers.^19^ Study methods are published elsewhere.^37^ Only adolescents aged 14 to 17 years were asked questions about lifetime spanking/slapping and child maltreatment, and therefore, this subsample was used. There were no significant differences in sex, household income, or single-parent status between this subsample of 14- to 17-year-olds compared to the remaining sample of 4- to 13-years-olds. Further restrictions included removing the small number of PMKs younger than 30 years (less than 0.6% of the sample). Demographic information indicated that these PMKs were not biological, step-, adoptive, or foster parents but rather another related male or female to the selected child, with an unspecified relationship. It was decided to exclude them, given that the demographic information and closeness in age to the selected child would indicate a greater likelihood of a sibling or other relative close in age acting as a caregiver, which could impact their “disciplinary” responses and experiences. The final sample size for this study was 1,883 adolescents. The privacy and confidentiality of participants were guaranteed under the Statistics Act. Informed consent was obtained from parents/caregivers and adolescents. Ethics approval for the study procedures for the 2014 OCHS was obtained from the Hamilton Integrated Research Ethics Board at McMaster University.

### Measurement

#### Lifetime spanking/slapping

Lifetime spanking/slapping was assessed by asking adolescents to self-report using a computerized questionnaire: “How many times did a parent and other caregivers spank you with their hand on your bottom (bum), or slapped you on your hand?” Response categories included *never, 1 or 2 times, 3 to 5 times, 6 to 10 times,* and *more than 10 times*. The variable was then dichotomized into 2 categories: 3 times or more versus 2 times or less. Similar to previous studies, spanking *1 or 2 times* was grouped with *never*, given that some parents/caregivers may spank their child once and choose to never do it again.^14^

#### Outcomes

Three outcome variables were studied including mental health disorders, physical health conditions, and defiant behaviors. Mental health disorders in the past 6 months were assessed in adolescents by interviewers using the Mini International Neuropsychiatric Interview for Children and Adolescents (MINI-KID), which yields sufficient estimates of reliability and validity.^38,39^ Scoring algorithms using screening questions and skip logic according to Diagnostic and Statistical Manual of Mental Disorders (DSM)-IV-TR criteria including impairment were used to create diagnosis classifications for 6 mental health disorders: (a) major depressive disorder; (b) separation anxiety disorder; (c) generalized and nongeneralized social phobia; (d) specific phobia; (e) inattentive, hyperactive, and combined attention deficit hyperactivity disorder (ADHD) subtypes; and (f) generalized anxiety disorder.^40^ Only adolescents who responded *yes*, *don’t know*, or *not stated* to the question “Has anyone—teacher, babysitter, friend or parent—ever complained about your behavior or about your performance in school?” were asked ADHD questions. An “any mental health disorder” variable was then created by combining the 6 mental health disorders into 1 dichotomous variable.

Physical health conditions were assessed by asking the parent/caregiver whether a health professional had ever diagnosed the adolescent with any of the following long-term conditions: (a) food or digestive allergies, (b) respiratory allergies such as hay fever, (c) any other allergies, (d) bronchitis, (e) diabetes, (f) heart condition or disease, (g) epilepsy, (h) cerebral palsy, (i) kidney condition or disease, (j) asthma, and (k) eczema. An “any physical health condition” variable was then created by combining the 11 physical health conditions into 1 dichotomous variable.

Similar to the mental disorder outcomes above, adolescent self-reported defiant behaviors were measured using the MINI-KID. With additional criteria, these symptoms can be categorized as a conduct disorder mental health diagnosis in the *DSM-IV*; however, it was examined as a separate outcome for 2 main reasons. First, this variable was used as a proxy for defiant behaviors that are problematic but not severe enough to meet diagnostic criteria. Second, spanking is often used by parents to “correct perceived misbehavior,” warranting a separate analysis for this behavioral outcome. Defiant behavior questions were only asked of a subset of adolescents who responded *yes*, *don’t know*, or *not stated* to the question “Has anyone—teacher, babysitter, friend or parent—ever complained about your behavior or about your performance in school?” A dichotomous “defiant behavior” variable was created based on the presence or absence of any of the following behaviors in the past year: (a) bullied or threatened other people, excluding brothers and sisters; (b) started fights with others, excluding brothers and sisters; (c) used a weapon to hurt someone, like a knife, gun, bat, or other object; (d) hurt someone, physically, on purpose, excluding brothers and sisters; (e) hurt animals on purpose; (f) stolen things using force, like robbing someone using a weapon or grabbing their handbag or wallet; (g) started fires on purpose in order to cause damage; (h) destroyed things that belonged to other people on purpose; (i) broken into someone’s house or car;(j) lied many times in order to get things from people or to get out of things; (k) tricked other people into doing what you wanted; (l) stolen things that were worth money, like shoplifting or stealing a credit card; (m) often stayed out a lot later than your parents let you; (n) run away from home two times or more; and (o) skipped school often.

#### Covariates

Covariates included sociodemographic variables (adolescent’s age, sex, household income, and single-parent status), the adolescents’ childhood adversities, and the adolescents’ experience of child maltreatment. A dichotomous variable of adolescents’ experiences of any childhood adversities was created, similar to the construct of ACEs, based on the presence or absence of any of the following experiences as reported by the parent/caregiver^26^: if the parent/caregiver ever (a) had problems with the use of alcohol or drugs; (b) broke the law repeatedly or did other things that could get them into trouble with the police; (c) talked to a doctor or counselor about problems with emotions, mental health, or use of alcohol or drugs; (d) were admitted for an overnight stay in a hospital or other facility to receive help for mental health problems or problems with alcohol or drugs; the child’s experience of (e) the death of the parent or sibling; and/or (f) separation or divorce of a parent.

Adolescents were asked about 5 types of child maltreatment including (a) physical abuse, (b) sexual abuse, (c) exposure to IPV (EIPV), (d) emotional abuse, and (e) physical neglect. Child maltreatment questions and cutoffs were derived from multiple sources including the Childhood Experiences of Violence Questionnaire,^41^ the National Longitudinal Study of Adolescent to Adult Health,^42^ and the Childhood Trauma Questionnaire.^43^ *Physical abuse* was present with any of the following experiences: an adult slapped them on the face, head, or ears or hit or spanked them with something hard to hurt them 3 times or more; pushed, grabbed, shoved, or threw something at them 3 times or more; and kicked, bit, punched, choked, burned, or physically attacked them in some way once or more. *Sexual abuse* was present with any of the following experiences: an adult forced them or attempted to force them into any unwanted sexual activity, by threatening them, holding them down, or hurting them in some way once or more and touched them against their will in any sexual way (anything from unwanted touching or grabbing to kissing or fondling) once or more. *EIPV* was present with any of the following experiences: parents or caregivers said hurtful or mean things to each other or to another adult in the home 6 times or more and heard any of them hit each other or another adult in the home 3 times or more. *Emotional abuse* was present when adolescents reported parents or caregivers said things that really hurt their feelings or made them feel like they were not wanted or loved 6 times or more.^43^ *Physical neglect* was present when youth reported parents or caregivers not taking care of their basic needs such as keeping them clean or providing food or clothing 1 time or more.^43^ An “any child maltreatment” variable was then created by combining the 5 types of child maltreatment into a dichotomous variable.

### Statistical Analysis

Analyses were conducted with Stata Version 15.0.^44^ Statistics Canada generated sampling weights that were applied in all analyses to adjust for selective sample losses and ensure its representativeness of the Ontario, Canada, population of households with children. Bootstrapping was used as a variance estimation method to produce standard errors and 95% confidence intervals (CI) with a Fay adjustment of 0.8 as per Statistics Canada’s user guidelines.^37,45^ The bootstrap weights, which take into account the complex sampling design to produce valid variance estimates, allowed for analyses conducted at the individual selected child level without clustering.^45^ Descriptive statistics were computed to examine the distribution of sociodemographic factors, childhood adversities, child maltreatment, and adolescent outcomes among those who had a lifetime history of being spanked/slapped. Logistic regression models were then computed to examine the association between slapping/spanking and the adolescent outcomes. Three logistic regression analyses were computed for each adolescent outcome: (1) unadjusted; (2) adjusting for adolescent’s sex, age, single parent, household income, and the adolescent’s childhood adversities; and (3) additionally adjusting for child maltreatment.

## Results

Table 1 presents the distribution of the adolescents’ sociodemographic factors, childhood adversities, child maltreatment, and adolescent outcomes among those who were spanked/slapped 3 or more times compared to 0 to 2 times in their lifetime. Previous research using the same sample found that 18% of adolescents aged 14 to 17 years had reported being spanked on the bottom and/or slapped on the hand 3 times or more in their lifetime.^19^ Information on missing data is available in Appendix A.

**Table 1.** Sociodemographic and Adolescent Outcomes by Lifetime History of Spanking/Slapping.

Measure	Spanking/Slapping 0 to 2 Times	Spanking/Slapping 3+ Times
Youth age (years)	15.5 (0.01)	15.7 (0.02)
Youth sex		
Males	83.6 (82.8 to 84.5)	16.4 (15.6 to 17.2)
Females	80.2 (79.2 to 81.3)	19.8 (18.7 to 20.8)
Household income (CA)		
$0 to $19,999	86.2 (84.2 to 87.9)	13.8 (12.1 to 15.8)
$20,000 to $39,999	85.4 (84.1 to 86.7)	14.6 (13.4 to 15.9)
$40,000 to $69,999	79.4 (77.8 to 81.0)	20.6 (19.0 to 22.3)
$70,000+	82.1 (81.3 to 82.9)	17.9 (17.1 to 18.7)
Single parent		
Two parent	82.3 (81.6 to 83.0)	17.7 (17.0 to 18.5)
Single parent	83.2 (81.7 to 84.7)	16.8 (15.4 to 18.3)
Childhood adversities		
No	85.0 (84.2 to 85.9)	15.0 (14.2 to 15.8)
Yes	79.2 (78.2 to 80.3)	20.8 (19.7 to 21.8)
Child maltreatment		
No	87.5 (86.7 to 88.2)	12.5 (11.8 to 13.3)
Yes	63.0 (61.4 to 64.6)	37.0 (35.4 to 38.6)
Mental health disorders		
No	84.6 (83.9 to 85.2)	15.4 (14.8 to 16.1)
Yes	71.5 (69.6 to 73.3)	28.5 (26.7 to 30.4)
Physical health conditions		
No	83.8 (83.0 to 84.6)	16.2 (15.5 to 17.0)
Yes	77.7 (76.4 to 79.0)	22.3 (21.0 to 23.6)
Defiant behaviors		
No	83.8 (82.1 to 85.4)	16.2 (14.6 to 17.9)
Yes	70.7 (68.9 to 72.5)	29.3 (27.5 to 31.2)

*Note.* Weighted prevalence and mean: % (95% confidence interval) or mean (standard error).

The results of the analyses examining the associations between lifetime experiences of being spanked/slapped and adolescent outcomes are presented in Table 2. Adolescents who experienced lifetime spanking/slapping 3 times or more had significantly increased odds of experiencing mental health disorders in adolescence (odds ratio [OR]: 2.19; 95% CI, 1.98 to 2.42). Although attenuated, the association remained statistically significant after adjusting for sociodemographic variables and childhood adversities (adjusted OR [AOR]-1: 1.57; 95% CI, 1.40 to 1.76) and further adjusting for child maltreatment (AOR-2: 1.29; 95% CI, 1.12 to 1.48). Experiencing spanking/slapping 3 times or more during the lifetime was significantly associated with physical health conditions in adolescence (OR: 1.48; 95% CI, 1.35 to 1.63). The association remained statistically significant after adjusting for sociodemographic variables and childhood adversities (AOR: 1.50; 95% CI, 1.36 to 1.65) and further adjusting for child maltreatment (AOR: 1.32; 95% CI, 1.20 to 1.45). Adolescents who were spanked/slapped 3 times or more during their lifetime had increased odds of demonstrating defiant behaviors in adolescence, and these associations were statistically significant in all three models (OR: 2.14; 95% CI, 1.84 to 2.50; AOR-1: 1.87; 95% CI, 1.59 to 2.20; and AOR-2: 1.93; 95% CI, 1.63 to 2.28). Trends and significance of all associations remained unchanged in post hoc sensitivity analyses with spanking coded as never (0 times) and any spanking (1+ times).

**Table 2.** Relationship between Lifetime History of Spanking/Slapping and Adolescent Outcomes.

Logistic regression model	Mental Disorders	Physical Health Conditions	Defiant Behaviors
OR (95% CI)	2.19 (1.98 to 2.42)***	1.48 (1.35 to 1.63)***	2.14 (1.84 to 2.50)***
AOR-1 (95% CI)	1.57 (1.40 to 1.76)***	1.50 (1.36 to 1.65)***	1.87 (1.59 to 2.20)***
AOR-2 (95% CI)	1.29 (1.12 to 1.48)***	1.32 (1.20 to 1.45)***	1.93 (1.63 to 2.28)***

*Note*. OR = Odds ratio; CI = confidence interval; AOR = adjusted odds ratio; AOR-1 = adjusting for child sex, child age, single parent, household income, and child’s childhood adversities. AOR-2 = adjusting for all covariates in AOR-1 with the addition of child maltreatment.

***P ≤ 0.001.

## Discussion

The current study involving a provincially representative sample of adolescents builds on past research by focusing specifically on outcomes associated with spanking/slapping.^2,14^ The findings demonstrate that these exposures are associated with mental health disorders, physical health conditions, and defiant behaviors independent of child maltreatment and other childhood adversities and are consistent with studies showing associations with “harsher” forms of physical punishment and similar outcomes.^21,24,34,36^ Childhood spanking has also been highly correlated with, and determined to be similar to, experiences of emotional abuse and physical abuse among an adult population.^14^ Together, these findings provide further empirical evidence to reject the prevailing belief that spanking/slapping is a distinct, “mild” form of physical punishment not associated with harmful outcomes.

Studying these associations with an adolescent sample is an important and unique strength of this study. Adolescence is a key developmental period characterized by a clustering of risk-taking behaviors, social and identity formation, a strong drive for independence and autonomy, challenging authority, new parenting challenges and concerns, and early onset and high prevalence of mental health disorders.^40,46–50^ Given the physical changes and psychosocial vulnerability of adolescence,^46,51,52^ it is possible that adolescents who have experienced lifetime spanking may be more susceptible to mental, physical, and behavioral challenges compared to those without such experiences.

Parents and other caregivers have endorsed the use of spanking/slapping because they believe in its effectiveness,^12,16^ including its perceived potential to *prevent* delinquency, aggression, and other behavioral challenges as well as to promote the development of moral internalization (i.e., the ability to learn right from wrong).^17,53,54^ Spanking/slapping is sometimes described as “corrective” in that it is believed that it corrects perceived misbehaviors. However, an important finding from this study is the significant association between lifetime spanking/slapping and *increased* defiant behaviors in adolescents after adjusting for other childhood adversities including child maltreatment—an important message for parents and caregivers to understand. While the cross-sectional nature of this study precludes making any causal inferences, the findings suggest that lifetime spanking/slapping does not *eliminate* delinquency, aggression, and other behavioral challenges, as intended by parents and caregivers who use such practices.^17,18,53^

It is important to take into consideration the limitations of the current study. First, as outlined above, causation cannot be inferred in the associations based on these cross-sectional data. It is unknown when the mental disorders, physical conditions, and defiant behaviors first manifested and whether they emerged prior to experiences of spanking/slapping. Similarly, the exact period of when spanking/slapping occurred is unknown. The possibility of a bidirectional relationship cannot be ruled out. Future longitudinal studies beginning in early childhood should be conducted to examine the relationship between spanking/slapping and adolescent outcomes. Second, given the retrospective nature of the data, the data may be subject to recall bias. For example, depending on the recency of spanking, adolescents may be more or less likely to recall such experiences. However, previous studies have demonstrated the reliability and validity of retrospective recall of childhood adversities including child maltreatment.^55,56^ Third, estimates of the youth’s physical health conditions were based on parent/caregiver reports of health professionals’ diagnoses. Confirmation of these reports with formal health-care provider diagnoses would improve the validity of the measure. However, previous research has found self-reported physical conditions to be valid and highly consistent with physician diagnoses.^57^ Fourth, due to low prevalence, we were unable to examine the associations between spanking/slapping and more specific mental health outcomes, physical health conditions, and defiant behaviors. It is possible that the associations with spanking/slapping might vary across specific conditions and behaviors. Fifth, while adjusting for childhood adversities is an important strength of this study, it is not an exhaustive list. Sixth, missing data may result in biased estimates.

This study provides further evidence that all forms of physical punishment, even perceived “acceptable” and “milder” forms such as spanking/slapping, are associated with harmful outcomes in adolescence including physical health problems, mental health disorders, and defiant behaviors. These findings can be used by adolescent and child-serving professionals and public health experts to further advocate for children’s rights to protection from violence including spanking/slapping and in particular in countries where physical punishment continues to be legal, prevalent, and widely accepted by parents and other caregivers.^8,11,12^ Together with existing literature, the current study provides additional empirical support in favor of eliminating and preventing spanking/slapping of all youth.

## Appendix A

### Missing Data

The percentage of missing data on each variable ranged from 0% to 14.7%: the largest percentages being on mental health disorders, any child maltreatment, and spanking. The percentage of respondents missing on at least 1 variable of interest was 24.8%; mental health disorders, any child maltreatment, and spanking accounted for the majority. There was an increased probability of missing on any variable for male compared to female adolescents. However, missing data did not differ by age, urbanicity, household income, single-parent status, or having seen a health-care provider because of concerns with the child’s mental health in the past 6 months.
